# Editorial on Optical Tweezers for the 15th Anniversary of Micromachines

**DOI:** 10.3390/mi15121522

**Published:** 2024-12-21

**Authors:** Mark Cronin-Golomb

**Affiliations:** Department of Biomedical Engineering, Tufts University, 4 Colby Street, Medford, MA 02155, USA; mark.cronin-golomb@tufts.edu

The electric fields of tightly focused laser beams can be strong enough to apply appreciable force to microscopic objects, including biological entities such as cells, bacteria, and even viruses and biomolecules [1,2]. Arthur Ashkin was the first to apply this effect to the trapping of micron-sized particles, both against a glass wall by a single beam and three dimensionally in suspension in aqueous solution by counterpropagating beams in 1970 [3]. In the mid-1980s, he and his co-workers showed that the optical gradient dipole force could overcome the scattering force to enable three-dimensional optical trapping by a single beam (optical tweezers) [4,5]. Since those discoveries, this research area has grown into a fully fledged field with over 17,000 research articles published since 1986 according to Clarivate’s Web of Science.

The reader might wonder whether an optical effect truly belongs in the scope of *Micromachines*. But considering that applications of micromachinery include the manipulation of objects at small scale, it becomes clear the answer is a definite yes. It joins a cohort of methods for the physical control of microparticles, especially in the context of biomedical applications. These include tweezers of various sorts, namely, mechanical [6,7], optical, magnetic [8,9,10], and acoustic [11,12]. In this editorial, I would like to consider how the field of optical tweezers has made its way firmly into the scope of the journal. A review by Zhu et al. [13] summarizes the many contributions made by optical tweezers to the field of optofluidics, an area pioneered in the early 2000s by Fainman and Psaltis [14,15]. As its name suggests, optofluidics represents a synergy between optics and microfluidics in which light is used for the sensing and control of materials in microchannel fluid handlers. Zhu’s review covers basic system design from optical force calculation to the application of holographic optical tweezers [16]. In another paper, Zhou et al. show how the standard Gerchberg–Saxton can be improved by adjusting the starting conditions to achieve faster convergence in the calculation of the patterns applied to a spatial light modulator for the generation of a desired array of optical traps [17]. Optical tweezers are often used to apply forces to biological cells, and the high thermal conductivity of the surrounding aqueous solution is often used to justify neglecting the optical damage potential. Experiments have shown that such an assumption may not be justified [18], and it may be advantageous to separate the optical intensity from the point of application of the force. One approach is to use indirect optical tweezing. The focused beams apply forces to micromachinery parts, such as polymer microspheres, or more complex constructs, which then transfer that force to the biological object being manipulated [19]. Chizari et al. have shown that it is possible to 3D-print the actuators via two-photon lithography and then to operate them using the same focused laser beam that was used to build the machine [20]. Sometimes it is difficult for an operator to effectively use these machines in a practical setting. Tanaka and Fujimoto have improved upon their previous designs for dual arm machines [21] to include haptic feedback using force feedback game controllers [22]. This addition enables the operator to sense, in real time, the size of force they are applying. Another recent paper concerns the use of optical tweezers in the generation and characterization of aerosol microdroplets. This technique was introduced by Hopkins et al. in 2004, who showed that microdroplets of water and decane can be held by a single-beam optical trap [23]. The recent paper by Li et al. provides some additional design parameters with respect to using relative humidity to control the formation of saline microdroplets in optical tweezers [24]. While there has been considerable progress in the formation of these microdroplets, more research is needed to enable their harvesting and subsequent use in applications.

The inclusion of the other forms of micro tweezers (magnetic, acoustic, mechanical) in the scope of this journal is valuable in that it encourages the cross fertilization of ideas. For example, in their paper on the use of texture-based (K, Na)NbO_3_ (KNN) ceramics for acoustic tweezers [25], Quan et al. use many of the same calculational beam propagation tools used for optical tweezers. Fabian et al. present a design for horizontal magnetic tweezers that involves separate three-axis micromanipulators for adjusting the relative positions of the sample and magnet [26], an idea that designers of optical tweezer devices might consider incorporating in their devices.

Scientific and engineering advances often depend on the adaptation of ideas from related areas that are sometimes isolated from each other by their publication in separate specialized journals. The incorporation of optical tweezers into the scope of *Micromachines* is a natural way to promote the productive cross-field dissemination of ideas.
